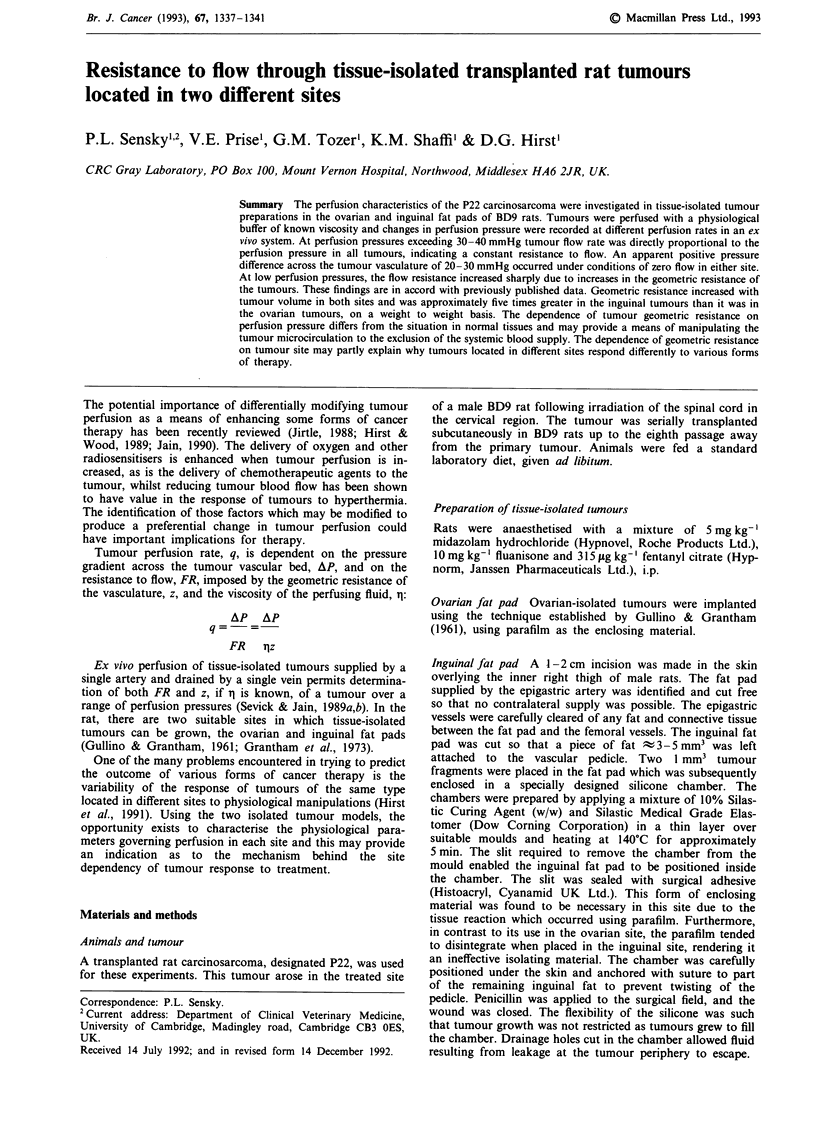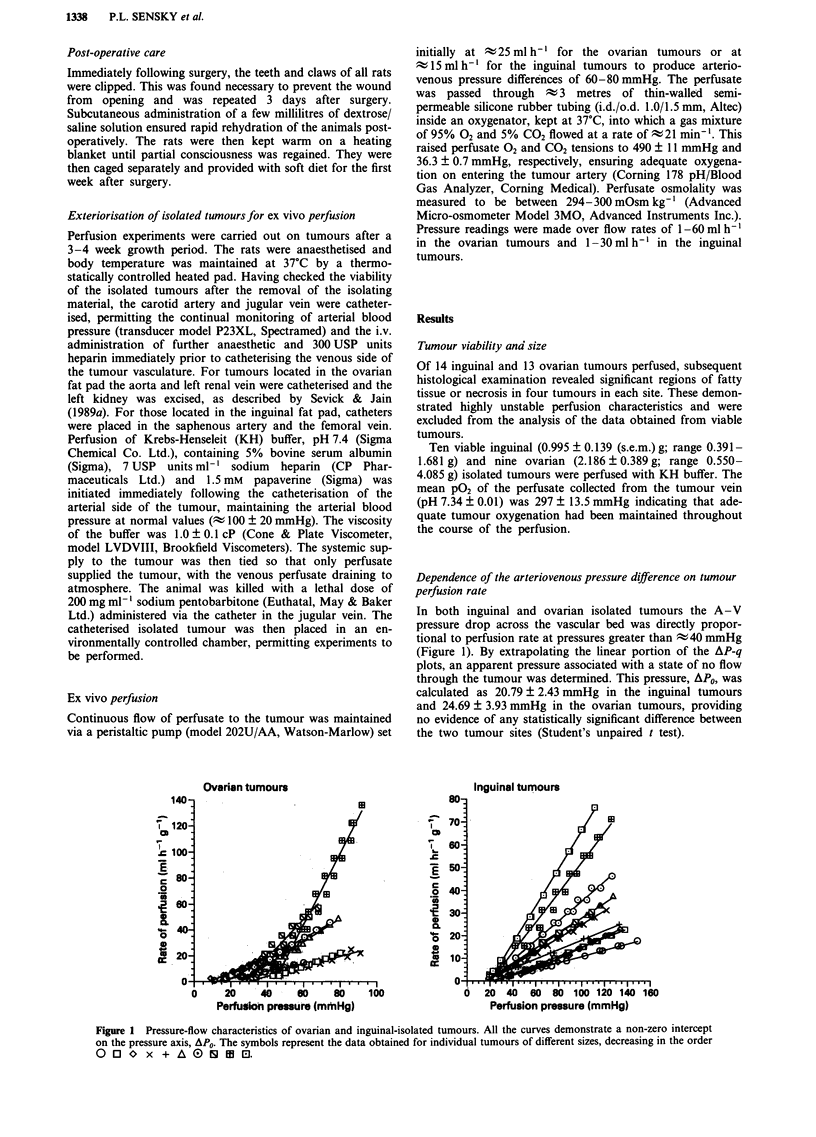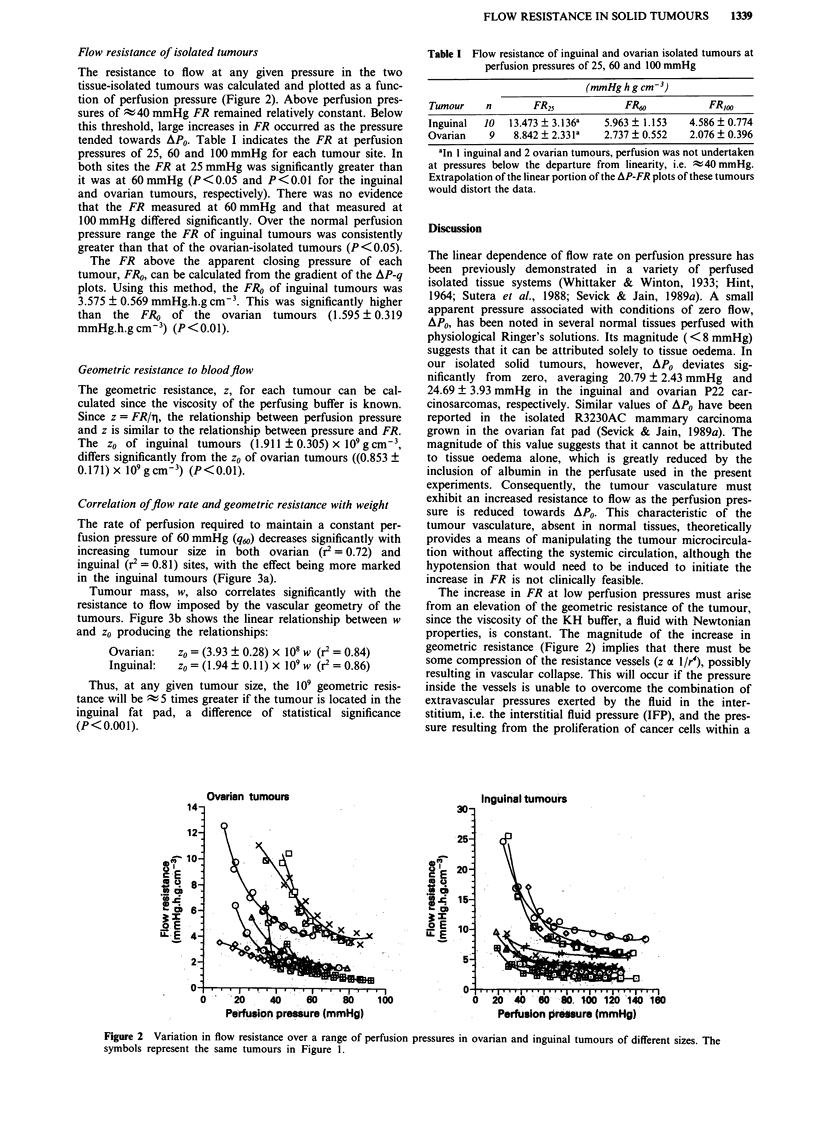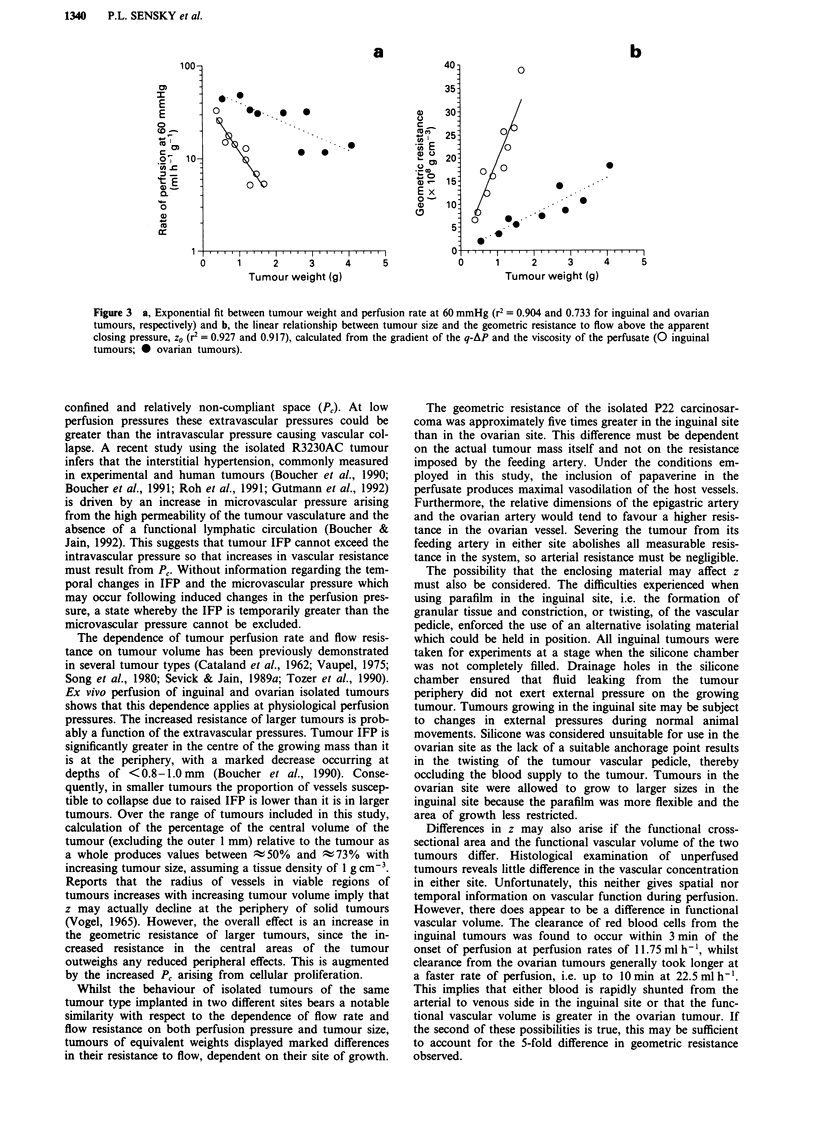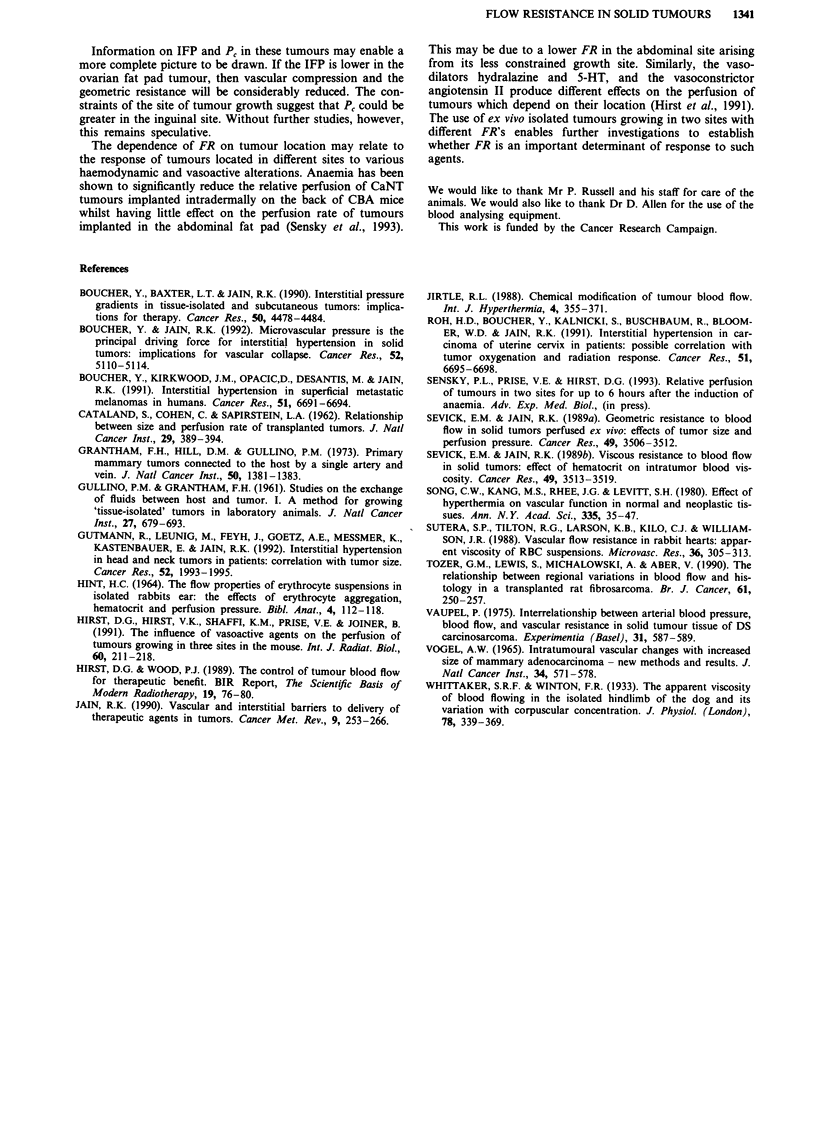# Resistance to flow through tissue-isolated transplanted rat tumours located in two different sites.

**DOI:** 10.1038/bjc.1993.247

**Published:** 1993-06

**Authors:** P. L. Sensky, V. E. Prise, G. M. Tozer, K. M. Shaffi, D. G. Hirst

**Affiliations:** CRC Gray Laboratory, Mount Vernon Hospital, Northwood, Middlesex, UK.

## Abstract

The perfusion characteristics of the P22 carcinosarcoma were investigated in tissue-isolated tumour preparations in the ovarian and inguinal fat pads of BD9 rats. Tumours were perfused with a physiological buffer of known viscosity and changes in perfusion pressure were recorded at different perfusion rates in an ex vivo system. At perfusion pressures exceeding 30-40 mmHg tumour flow rate was directly proportional to the perfusion pressure in all tumours, indicating a constant resistance to flow. An apparent positive pressure difference across the tumour vasculature of 20-30 mmHg occurred under conditions of zero flow in either site. At low perfusion pressures, the flow resistance increased sharply due to increases in the geometric resistance of the tumours. These findings are in accord with previously published data. Geometric resistance increased with tumour volume in both sites and was approximately five times greater in the inguinal tumours than it was in the ovarian tumours, on a weight to weight basis. The dependence of tumour geometric resistance on perfusion pressure differs from the situation in normal tissues and may provide a means of manipulating the tumour microcirculation to the exclusion of the systemic blood supply. The dependence of geometric resistance on tumour site may partly explain why tumours located in different sites respond differently to various forms of therapy.


Br. J. Cancer (1993), 67, 1337-1341 © Macmillan Press Ltd., 1993

### Resistance to flow through tissue-isolated transplanted rat tumours located in two different sites

P.L. Sensky[1,2], V.E. Prise[1], G.M. Tozer[1], K.M. Shaffi[1] & D.G. Hirst[1]

*CRC Gray Laboratory, PO Box 100, Mount Vernon Hospital, Northwood, Middlesex HA6 2JR, UK.*

**Summary** The perfusion characteristics of the P22 carcinosarcoma were investigated in tissue-isolated tumour preparations in the ovarian and inguinal fat pads of BD9 rats. Tumours were perfused with a physiological buffer of known viscosity and changes in perfusion pressure were recorded at different perfusion rates in an *ex vivo* system. At perfusion pressures exceeding 30-40 mmHg tumour flow rate was directly proportional to the perfusion pressure in all tumours, indicating a constant resistance to flow. An apparent positive pressure difference across the tumour vasculature of 20-30 mmHg occurred under conditions of zero flow in either site. At low perfusion pressures, the flow resistance increased sharply due to increases in the geometric resistance of the tumours. These findings are in accord with previously published data. Geometric resistance increased with tumour volume in both sites and was approximately five times greater in the inguinal tumours than it was in the ovarian tumours, on a weight to weight basis. The dependence of tumour geometric resistance on perfusion pressure differs from the situation in normal tissues and may provide a means of manipulating the tumour microcirculation to the exclusion of the systemic blood supply. The dependence of geometric resistance on tumour site may partly explain why tumours located in different sites respond differently to various forms of therapy.

The potential importance of differentially modifying tumour perfusion as a means of enhancing some forms of cancer therapy has been recently reviewed (Jirtle, 1988; Hirst & Wood, 1989; Jain, 1990). The delivery of oxygen and other radiosensitisers is enhanced when tumour perfusion is increased, as is the delivery of chemotherapeutic agents to the tumour, whilst reducing tumour blood flow has been shown to have value in the response of tumours to hyperthermia. The identification of those factors which may be modified to produce a preferential change in tumour perfusion could have important implications for therapy.

Tumour perfusion rate, $q$, is dependent on the pressure gradient across the tumour vascular bed, $\Delta P$, and on the resistance to flow, $FR$, imposed by the geometric resistance of the vasculature, $z$, and the viscosity of the perfusing fluid, $\eta$:

$$q = \frac{\Delta P}{FR} = \frac{\Delta P}{\eta z}$$

*Ex vivo* perfusion of tissue-isolated tumours supplied by a single artery and drained by a single vein permits determination of both $FR$ and $z$, if $\eta$ is known, of a tumour over a range of perfusion pressures (Sevick & Jain, 1989*a,b*). In the rat, there are two suitable sites in which tissue-isolated tumours can be grown, the ovarian and inguinal fat pads (Gullino & Grantham, 1961; Grantham *et al.*, 1973).

One of the many problems encountered in trying to predict the outcome of various forms of cancer therapy is the variability of the response of tumours of the same type located in different sites to physiological manipulations (Hirst *et al.*, 1991). Using the two isolated tumour models, the opportunity exists to characterise the physiological parameters governing perfusion in each site and this may provide an indication as to the mechanism behind the site dependency of tumour response to treatment.

### Materials and methods

### Animals and tumour

A transplanted rat carcinosarcoma, designated P22, was used for these experiments. This tumour arose in the treated site of a male BD9 rat following irradiation of the spinal cord in the cervical region. The tumour was serially transplanted subcutaneously in BD9 rats up to the eighth passage away from the primary tumour. Animals were fed a standard laboratory diet, given *ad libitum*.

### Preparation of tissue-isolated tumours

Rats were anaesthetised with a mixture of 5 mg kg$^{-1}$ midazolam hydrochloride (Hypnovel, Roche Products Ltd.), 10 mg kg$^{-1}$ fluanisone and 315 $\mu$g kg$^{-1}$ fentanyl citrate (Hypnorm, Janssen Pharmaceuticals Ltd.), i.p.

*Ovarian fat pad* Ovarian-isolated tumours were implanted using the technique established by Gullino & Grantham (1961), using parafilm as the enclosing material.

*Inguinal fat pad* A 1-2 cm incision was made in the skin overlying the inner right thigh of male rats. The fat pad supplied by the epigastric artery was identified and cut free so that no contralateral supply was possible. The epigastric vessels were carefully cleared of any fat and connective tissue between the fat pad and the femoral vessels. The inguinal fat pad was cut so that a piece of fat $\approx$3-5 mm$^3$ was left attached to the vascular pedicle. Two 1 mm$^3$ tumour fragments were placed in the fat pad which was subsequently enclosed in a specially designed silicone chamber. The chambers were prepared by applying a mixture of 10% Silastic Curing Agent (w/w) and Silastic Medical Grade Elastomer (Dow Corning Corporation) in a thin layer over suitable moulds and heating at 140°C for approximately 5 min. The slit required to remove the chamber from the mould enabled the inguinal fat pad to be positioned inside the chamber. The slit was sealed with surgical adhesive (Histoacryl, Cyanamid UK Ltd.). This form of enclosing material was found to be necessary in this site due to the tissue reaction which occurred using parafilm. Furthermore, in contrast to its use in the ovarian site, the parafilm tended to disintegrate when placed in the inguinal site, rendering it an ineffective isolating material. The chamber was carefully positioned under the skin and anchored with suture to part of the remaining inguinal fat to prevent twisting of the pedicle. Penicillin was applied to the surgical field, and the wound was closed. The flexibility of the silicone was such that tumour growth was not restricted as tumours grew to fill the chamber. Drainage holes cut in the chamber allowed fluid resulting from leakage at the tumour periphery to escape.

Correspondence: P.L. Sensky.
[2]Current address: Department of Clinical Veterinary Medicine, University of Cambridge, Madingley road, Cambridge CB3 0ES, UK.
Received 14 July 1992; and in revised form 14 December 1992.

*Post-operative care*

Immediately following surgery, the teeth and claws of all rats were clipped. This was found necessary to prevent the wound from opening and was repeated 3 days after surgery. Subcutaneous administration of a few millilitres of dextrose/saline solution ensured rapid rehydration of the animals post-operatively. The rats were then kept warm on a heating blanket until partial consciousness was regained. They were then caged separately and provided with soft diet for the first week after surgery.

*Exteriorisation of isolated tumours for* ex vivo *perfusion*

Perfusion experiments were carried out on tumours after a 3–4 week growth period. The rats were anaesthetised and body temperature was maintained at 37°C by a thermostatically controlled heated pad. Having checked the viability of the isolated tumours after the removal of the isolating material, the carotid artery and jugular vein were catheterised, permitting the continual monitoring of arterial blood pressure (transducer model P23XL, Spectramed) and the i.v. administration of further anaesthetic and 300 USP units heparin immediately prior to catheterising the venous side of the tumour vasculature. For tumours located in the ovarian fat pad the aorta and left renal vein were catheterised and the left kidney was excised, as described by Sevick & Jain (1989*a*). For those located in the inguinal fat pad, catheters were placed in the saphenous artery and the femoral vein. Perfusion of Krebs-Henseleit (KH) buffer, pH 7.4 (Sigma Chemical Co. Ltd.), containing 5% bovine serum albumin (Sigma), 7 USP units $ml^{-1}$ sodium heparin (CP Pharmaceuticals Ltd.) and 1.5 mM papaverine (Sigma) was initiated immediately following the catheterisation of the arterial side of the tumour, maintaining the arterial blood pressure at normal values ($\approx 100 \pm 20$ mmHg). The viscosity of the buffer was $1.0 \pm 0.1$ cP (Cone & Plate Viscometer, model LVDVIII, Brookfield Viscometers). The systemic supply to the tumour was then tied so that only perfusate supplied the tumour, with the venous perfusate draining to atmosphere. The animal was killed with a lethal dose of 200 mg $ml^{-1}$ sodium pentobarbitone (Euthatal, May & Baker Ltd.) administered via the catheter in the jugular vein. The catheterised isolated tumour was then placed in an environmentally controlled chamber, permitting experiments to be performed.

Ex vivo *perfusion*

Continuous flow of perfusate to the tumour was maintained via a peristaltic pump (model 202U/AA, Watson-Marlow) set initially at $\approx 25$ ml $h^{-1}$ for the ovarian tumours or at $\approx 15$ ml $h^{-1}$ for the inguinal tumours to produce arteriovenous pressure differences of 60–80 mmHg. The perfusate was passed through $\approx 3$ metres of thin-walled semi-permeable silicone rubber tubing (i.d./o.d. 1.0/1.5 mm, Altec) inside an oxygenator, kept at 37°C, into which a gas mixture of 95% $O_2$ and 5% $CO_2$ flowed at a rate of $\approx 2$ l $min^{-1}$. This raised perfusate $O_2$ and $CO_2$ tensions to $490 \pm 11$ mmHg and $36.3 \pm 0.7$ mmHg, respectively, ensuring adequate oxygenation on entering the tumour artery (Corning 178 pH/Blood Gas Analyzer, Corning Medical). Perfusate osmolality was measured to be between 294–300 mOsm $kg^{-1}$ (Advanced Micro-osmometer Model 3MO, Advanced Instruments Inc.). Pressure readings were made over flow rates of 1–60 ml $h^{-1}$ in the ovarian tumours and 1–30 ml $h^{-1}$ in the inguinal tumours.

### Results

*Tumour viability and size*

Of 14 inguinal and 13 ovarian tumours perfused, subsequent histological examination revealed significant regions of fatty tissue or necrosis in four tumours in each site. These demonstrated highly unstable perfusion characteristics and were excluded from the analysis of the data obtained from viable tumours.

Ten viable inguinal ($0.995 \pm 0.139$ (s.e.m.) g; range 0.391–1.681 g) and nine ovarian ($2.186 \pm 0.389$ g; range 0.550–4.085 g) isolated tumours were perfused with KH buffer. The mean $pO_2$ of the perfusate collected from the tumour vein (pH $7.34 \pm 0.01$) was $297 \pm 13.5$ mmHg indicating that adequate tumour oxygenation had been maintained throughout the course of the perfusion.

*Dependence of the arteriovenous pressure difference on tumour perfusion rate*

In both inguinal and ovarian isolated tumours the A–V pressure drop across the vascular bed was directly proportional to perfusion rate at pressures greater than $\approx 40$ mmHg (Figure 1). By extrapolating the linear portion of the $\Delta P$-$q$ plots, an apparent pressure associated with a state of no flow through the tumour was determined. This pressure, $\Delta P_0$, was calculated as $20.79 \pm 2.43$ mmHg in the inguinal tumours and $24.69 \pm 3.93$ mmHg in the ovarian tumours, providing no evidence of any statistically significant difference between the two tumour sites (Student's unpaired $t$ test).

**Figure 1** Pressure-flow characteristics of ovarian and inguinal-isolated tumours. All the curves demonstrate a non-zero intercept on the pressure axis, $\Delta P_0$. The symbols represent the data obtained for individual tumours of different sizes, decreasing in the order ○ □ ◇ × + △ ⊙ ⊠ ⊞ ⊡.

### *Flow resistance of isolated tumours*

The resistance to flow at any given pressure in the two tissue-isolated tumours was calculated and plotted as a function of perfusion pressure (Figure 2). Above perfusion pressures of ≈40 mmHg *FR* remained relatively constant. Below this threshold, large increases in *FR* occurred as the pressure tended towards $\Delta P_0$. Table I indicates the *FR* at perfusion pressures of 25, 60 and 100 mmHg for each tumour site. In both sites the *FR* at 25 mmHg was significantly greater than it was at 60 mmHg ($P<0.05$ and $P<0.01$ for the inguinal and ovarian tumours, respectively). There was no evidence that the *FR* measured at 60 mmHg and that measured at 100 mmHg differed significantly. Over the normal perfusion pressure range the *FR* of inguinal tumours was consistently greater than that of the ovarian-isolated tumours ($P<0.05$).

The *FR* above the apparent closing pressure of each tumour, $FR_0$, can be calculated from the gradient of the $\Delta P$-$q$ plots. Using this method, the $FR_0$ of inguinal tumours was $3.575 \pm 0.569$ mmHg.h.g cm$^{-3}$. This was significantly higher than the $FR_0$ of the ovarian tumours ($1.595 \pm 0.319$ mmHg.h.g cm$^{-3}$) ($P<0.01$).

### *Geometric resistance to blood flow*

The geometric resistance, $z$, for each tumour can be calculated since the viscosity of the perfusing buffer is known. Since $z = FR/\eta$, the relationship between perfusion pressure and $z$ is similar to the relationship between pressure and *FR*. The $z_0$ of inguinal tumours $(1.911 \pm 0.305) \times 10^9$ g cm$^{-3}$, differs significantly from the $z_0$ of ovarian tumours ($(0.853 \pm 0.171) \times 10^9$ g cm$^{-3}$) ($P<0.01$).

### *Correlation of flow rate and geometric resistance with weight*

The rate of perfusion required to maintain a constant perfusion pressure of 60 mmHg ($q_{60}$) decreases significantly with increasing tumour size in both ovarian ($r^2 = 0.72$) and inguinal ($r^2 = 0.81$) sites, with the effect being more marked in the inguinal tumours (Figure 3a).

Tumour mass, $w$, also correlates significantly with the resistance to flow imposed by the vascular geometry of the tumours. Figure 3b shows the linear relationship between $w$ and $z_0$ producing the relationships:

Ovarian: $z_0 = (3.93 \pm 0.28) \times 10^8\, w$ ($r^2 = 0.84$)

Inguinal: $z_0 = (1.94 \pm 0.11) \times 10^9\, w$ ($r^2 = 0.86$)

Thus, at any given tumour size, the $10^9$ geometric resistance will be ≈5 times greater if the tumour is located in the inguinal fat pad, a difference of statistical significance ($P<0.001$).

**Table I** Flow resistance of inguinal and ovarian isolated tumours at perfusion pressures of 25, 60 and 100 mmHg

| | | (mmHg h g cm$^{-3}$) | | |
|---|---|---|---|---|
| *Tumour* | *n* | $FR_{25}$ | $FR_{60}$ | $FR_{100}$ |
| Inguinal | *10* | 13.473 ± 3.136[a] | 5.963 ± 1.153 | 4.586 ± 0.774 |
| Ovarian | *9* | 8.842 ± 2.331[a] | 2.737 ± 0.552 | 2.076 ± 0.396 |

[a]In 1 inguinal and 2 ovarian tumours, perfusion was not undertaken at pressures below the departure from linearity, i.e. ≈40 mmHg. Extrapolation of the linear portion of the $\Delta P$-*FR* plots of these tumours would distort the data.

### Discussion

The linear dependence of flow rate on perfusion pressure has been previously demonstrated in a variety of perfused isolated tissue systems (Whittaker & Winton, 1933; Hint, 1964; Sutera *et al.*, 1988; Sevick & Jain, 1989*a*). A small apparent pressure associated with conditions of zero flow, $\Delta P_0$, has been noted in several normal tissues perfused with physiological Ringer's solutions. Its magnitude (<8 mmHg) suggests that it can be attributed solely to tissue oedema. In our isolated solid tumours, however, $\Delta P_0$ deviates significantly from zero, averaging $20.79 \pm 2.43$ mmHg and $24.69 \pm 3.93$ mmHg in the inguinal and ovarian P22 carcinosarcomas, respectively. Similar values of $\Delta P_0$ have been reported in the isolated R3230AC mammary carcinoma grown in the ovarian fat pad (Sevick & Jain, 1989*a*). The magnitude of this value suggests that it cannot be attributed to tissue oedema alone, which is greatly reduced by the inclusion of albumin in the perfusate used in the present experiments. Consequently, the tumour vasculature must exhibit an increased resistance to flow as the perfusion pressure is reduced towards $\Delta P_0$. This characteristic of the tumour vasculature, absent in normal tissues, theoretically provides a means of manipulating the tumour microcirculation without affecting the systemic circulation, although the hypotension that would need to be induced to initiate the increase in *FR* is not clinically feasible.

The increase in *FR* at low perfusion pressures must arise from an elevation of the geometric resistance of the tumour, since the viscosity of the KH buffer, a fluid with Newtonian properties, is constant. The magnitude of the increase in geometric resistance (Figure 2) implies that there must be some compression of the resistance vessels ($z \propto 1/r^4$), possibly resulting in vascular collapse. This will occur if the pressure inside the vessels is unable to overcome the combination of extravascular pressures exerted by the fluid in the interstitium, i.e. the interstitial fluid pressure (IFP), and the pressure resulting from the proliferation of cancer cells within a

**Figure 2** Variation in flow resistance over a range of perfusion pressures in ovarian and inguinal tumours of different sizes. The symbols represent the same tumours in Figure 1.

**Figure 3** **a**, Exponential fit between tumour weight and perfusion rate at 60 mmHg ($r^2 = 0.904$ and 0.733 for inguinal and ovarian tumours, respectively) and **b**, the linear relationship between tumour size and the geometric resistance to flow above the apparent closing pressure, $z_0$ ($r^2 = 0.927$ and 0.917), calculated from the gradient of the $q$-$\Delta P$ and the viscosity of the perfusate (○ inguinal tumours; ● ovarian tumours).

confined and relatively non-compliant space ($P_c$). At low perfusion pressures these extravascular pressures could be greater than the intravascular pressure causing vascular collapse. A recent study using the isolated R3230AC tumour infers that the interstitial hypertension, commonly measured in experimental and human tumours (Boucher *et al.*, 1990; Boucher *et al.*, 1991; Roh *et al.*, 1991; Gutmann *et al.*, 1992) is driven by an increase in microvascular pressure arising from the high permeability of the tumour vasculature and the absence of a functional lymphatic circulation (Boucher & Jain, 1992). This suggests that tumour IFP cannot exceed the intravascular pressure so that increases in vascular resistance must result from $P_c$. Without information regarding the temporal changes in IFP and the microvascular pressure which may occur following induced changes in the perfusion pressure, a state whereby the IFP is temporarily greater than the microvascular pressure cannot be excluded.

The dependence of tumour perfusion rate and flow resistance on tumour volume has been previously demonstrated in several tumour types (Cataland *et al.*, 1962; Vaupel, 1975; Song *et al.*, 1980; Sevick & Jain, 1989*a*; Tozer *et al.*, 1990). *Ex vivo* perfusion of inguinal and ovarian isolated tumours shows that this dependence applies at physiological perfusion pressures. The increased resistance of larger tumours is probably a function of the extravascular pressures. Tumour IFP is significantly greater in the centre of the growing mass than it is at the periphery, with a marked decrease occurring at depths of $<0.8-1.0$ mm (Boucher *et al.*, 1990). Consequently, in smaller tumours the proportion of vessels susceptible to collapse due to raised IFP is lower than it is in larger tumours. Over the range of tumours included in this study, calculation of the percentage of the central volume of the tumour (excluding the outer 1 mm) relative to the tumour as a whole produces values between ≈50% and ≈73% with increasing tumour size, assuming a tissue density of 1 g cm$^{-3}$. Reports that the radius of vessels in viable regions of tumours increases with increasing tumour volume imply that $z$ may actually decline at the periphery of solid tumours (Vogel, 1965). However, the overall effect is an increase in the geometric resistance of larger tumours, since the increased resistance in the central areas of the tumour outweighs any reduced peripheral effects. This is augmented by the increased $P_c$ arising from cellular proliferation.

Whilst the behaviour of isolated tumours of the same tumour type implanted in two different sites bears a notable similarity with respect to the dependence of flow rate and flow resistance on both perfusion pressure and tumour size, tumours of equivalent weights displayed marked differences in their resistance to flow, dependent on their site of growth.

The geometric resistance of the isolated P22 carcinosarcoma was approximately five times greater in the inguinal site than in the ovarian site. This difference must be dependent on the actual tumour mass itself and not on the resistance imposed by the feeding artery. Under the conditions employed in this study, the inclusion of papaverine in the perfusate produces maximal vasodilation of the host vessels. Furthermore, the relative dimensions of the epigastric artery and the ovarian artery would tend to favour a higher resistance in the ovarian vessel. Severing the tumour from its feeding artery in either site abolishes all measurable resistance in the system, so arterial resistance must be negligible.

The possibility that the enclosing material may affect $z$ must also be considered. The difficulties experienced when using parafilm in the inguinal site, i.e. the formation of granular tissue and constriction, or twisting, of the vascular pedicle, enforced the use of an alternative isolating material which could be held in position. All inguinal tumours were taken for experiments at a stage when the silicone chamber was not completely filled. Drainage holes in the silicone chamber ensured that fluid leaking from the tumour periphery did not exert external pressure on the growing tumour. Tumours growing in the inguinal site may be subject to changes in external pressures during normal animal movements. Silicone was considered unsuitable for use in the ovarian site as the lack of a suitable anchorage point results in the twisting of the tumour vascular pedicle, thereby occluding the blood supply to the tumour. Tumours in the ovarian site were allowed to grow to larger sizes in the inguinal site because the parafilm was more flexible and the area of growth less restricted.

Differences in $z$ may also arise if the functional cross-sectional area and the functional vascular volume of the two tumours differ. Histological examination of unperfused tumours reveals little difference in the vascular concentration in either site. Unfortunately, this neither gives spatial nor temporal information on vascular function during perfusion. However, there does appear to be a difference in functional vascular volume. The clearance of red blood cells from the inguinal tumours was found to occur within 3 min of the onset of perfusion at perfusion rates of 11.75 ml h$^{-1}$, whilst clearance from the ovarian tumours generally took longer at a faster rate of perfusion, i.e. up to 10 min at 22.5 ml h$^{-1}$. This implies that either blood is rapidly shunted from the arterial to venous side in the inguinal site or that the functional vascular volume is greater in the ovarian tumour. If the second of these possibilities is true, this may be sufficient to account for the 5-fold difference in geometric resistance observed.

Information on IFP and $P_c$ in these tumours may enable a more complete picture to be drawn. If the IFP is lower in the ovarian fat pad tumour, then vascular compression and the geometric resistance will be considerably reduced. The constraints of the site of tumour growth suggest that $P_c$ could be greater in the inguinal site. Without further studies, however, this remains speculative.

The dependence of *FR* on tumour location may relate to the response of tumours located in different sites to various haemodynamic and vasoactive alterations. Anaemia has been shown to significantly reduce the relative perfusion of CaNT tumours implanted intradermally on the back of CBA mice whilst having little effect on the perfusion rate of tumours implanted in the abdominal fat pad (Sensky *et al.*, 1993). This may be due to a lower *FR* in the abdominal site arising from its less constrained growth site. Similarly, the vasodilators hydralazine and 5-HT, and the vasoconstrictor angiotensin II produce different effects on the perfusion of tumours which depend on their location (Hirst *et al.*, 1991). The use of *ex vivo* isolated tumours growing in two sites with different *FR*'s enables further investigations to establish whether *FR* is an important determinant of response to such agents.

We would like to thank Mr P. Russell and his staff for care of the animals. We would also like to thank Dr D. Allen for the use of the blood analysing equipment.

This work is funded by the Cancer Research Campaign.

### References

BOUCHER, Y., BAXTER, L.T. & JAIN, R.K. (1990). Interstitial pressure gradients in tissue-isolated and subcutaneous tumors: implications for therapy. *Cancer Res.*, **50**, 4478–4484.

BOUCHER, Y. & JAIN, R.K. (1992). Microvascular pressure is the principal driving force for interstitial hypertension in solid tumors: implications for vascular collapse. *Cancer Res.*, **52**, 5110–5114.

BOUCHER, Y., KIRKWOOD, J.M., OPACIC,D., DESANTIS, M. & JAIN, R.K. (1991). Interstitial hypertension in superficial metastatic melanomas in humans. *Cancer Res.*, **51**, 6691–6694.

CATALAND, S., COHEN, C. & SAPIRSTEIN, L.A. (1962). Relationship between size and perfusion rate of transplanted tumors. *J. Natl Cancer Inst.*, **29**, 389–394.

GRANTHAM, F.H., HILL, D.M. & GULLINO, P.M. (1973). Primary mammary tumors connected to the host by a single artery and vein. *J. Natl Cancer Inst.*, **50**, 1381–1383.

GULLINO, P.M. & GRANTHAM, F.H. (1961). Studies on the exchange of fluids between host and tumor. I. A method for growing 'tissue-isolated' tumors in laboratory animals. *J. Natl Cancer Inst.*, **27**, 679–693.

GUTMANN, R., LEUNIG, M., FEYH, J., GOETZ, A.E., MESSMER, K., KASTENBAUER, E. & JAIN, R.K. (1992). Interstitial hypertension in head and neck tumors in patients: correlation with tumor size. *Cancer Res.*, **52**, 1993–1995.

HINT, H.C. (1964). The flow properties of erythrocyte suspensions in isolated rabbits ear: the effects of erythrocyte aggregation, hematocrit and perfusion pressure. *Bibl. Anat.*, **4**, 112–118.

HIRST, D.G., HIRST, V.K., SHAFFI, K.M., PRISE, V.E. & JOINER, B. (1991). The influence of vasoactive agents on the perfusion of tumours growing in three sites in the mouse. *Int. J. Radiat. Biol.*, **60**, 211–218.

HIRST, D.G. & WOOD, P.J. (1989). The control of tumour blood flow for therapeutic benefit. BIR Report, *The Scientific Basis of Modern Radiotherapy*, **19**, 76–80.

JAIN, R.K. (1990). Vascular and interstitial barriers to delivery of therapeutic agents in tumors. *Cancer Met. Rev.*, **9**, 253–266.

JIRTLE, R.L. (1988). Chemical modification of tumour blood flow. *Int. J. Hyperthermia*, **4**, 355–371.

ROH, H.D., BOUCHER, Y., KALNICKI, S., BUSCHBAUM, R., BLOOMER, W.D. & JAIN, R.K. (1991). Interstitial hypertension in carcinoma of uterine cervix in patients: possible correlation with tumor oxygenation and radiation response. *Cancer Res.*, **51**, 6695–6698.

SENSKY, P.L., PRISE, V.E. & HIRST, D.G. (1993). Relative perfusion of tumours in two sites for up to 6 hours after the induction of anaemia. *Adv. Exp. Med. Biol.*, (in press).

SEVICK, E.M. & JAIN, R.K. (1989*a*). Geometric resistance to blood flow in solid tumors perfused *ex vivo*: effects of tumor size and perfusion pressure. *Cancer Res.*, **49**, 3506–3512.

SEVICK, E.M. & JAIN, R.K. (1989*b*). Viscous resistance to blood flow in solid tumors: effect of hematocrit on intratumor blood viscosity. *Cancer Res.*, **49**, 3513–3519.

SONG, C.W., KANG, M.S., RHEE, J.G. & LEVITT, S.H. (1980). Effect of hyperthermia on vascular function in normal and neoplastic tissues. *Ann. N.Y. Acad. Sci.*, **335**, 35–47.

SUTERA, S.P., TILTON, R.G., LARSON, K.B., KILO, C.J. & WILLIAMSON, J.R. (1988). Vascular flow resistance in rabbit hearts: apparent viscosity of RBC suspensions. *Microvasc. Res.*, **36**, 305–313.

TOZER, G.M., LEWIS, S., MICHALOWSKI, A. & ABER, V. (1990). The relationship between regional variations in blood flow and histology in a transplanted rat fibrosarcoma. *Br. J. Cancer*, **61**, 250–257.

VAUPEL, P. (1975). Interrelationship between arterial blood pressure, blood flow, and vascular resistance in solid tumour tissue of DS carcinosarcoma. *Experimentia (Basel)*, **31**, 587–589.

VOGEL, A.W. (1965). Intratumoural vascular changes with increased size of mammary adenocarcinoma – new methods and results. *J. Natl Cancer Inst.*, **34**, 571–578.

WHITTAKER, S.R.F. & WINTON, F.R. (1933). The apparent viscosity of blood flowing in the isolated hindlimb of the dog and its variation with corpuscular concentration. *J. Physiol. (London)*, **78**, 339–369.